# A different view on fine-scale population structure in Western African populations

**DOI:** 10.1007/s00439-019-02069-7

**Published:** 2019-10-19

**Authors:** Kridsadakorn Chaichoompu, Fentaw Abegaz, Bruno Cavadas, Verónica Fernandes, Bertram Müller-Myhsok, Luísa Pereira, Kristel Van Steen

**Affiliations:** 1 GIGA-R Medical Genomics-BIO3, University of Liege, Avenue de l’Hôpital 11, 4000 Liege, Belgium; 2 Max Planck Institute of Psychiatry, 80804 Munich, Germany; 3 Instituto de Investigação e Inovação em Saúde, Universidade do Porto (i3S), Rua Alfredo Allen, 208, 4200-135 Porto, Portugal; 4 Instituto de Patologia e Imunologia Molecular da Universidade do Porto (IPATIMUP), Rua Júlio Amaral de Carvalho, 45, 4200-135 Porto, Portugal; 5 WELBIO (Walloon Excellence in Lifesciences and Biotechnology), Avenue Pasteur 6, 1300 Wavre, Belgium

## Abstract

Due to its long genetic evolutionary history, Africans exhibit more genetic variation than any other population in the world. Their genetic diversity further lends itself to subdivisions of Africans into groups of individuals with a genetic similarity of varying degrees of granularity. It remains challenging to detect fine-scale structure in a computationally efficient and meaningful way. In this paper, we present a proof-of-concept of a novel fine-scale population structure detection tool with Western African samples. These samples consist of 1396 individuals from 25 ethnic groups (two groups are African American descendants). The strategy is based on a recently developed tool called IPCAPS. IPCAPS, or Iterative Pruning to CApture Population Structure, is a genetic divisive clustering strategy that enhances iterative pruning PCA, is robust to outliers and does not require a priori computation of haplotypes. Our strategy identified in total 12 groups and 6 groups were revealed as fine-scale structure detected in the samples from Cameroon, Gambia, Mali, Southwest USA, and Barbados. Our finding helped to explain evolutionary processes in the analyzed West African samples and raise awareness for fine-scale structure resolution when conducting genome-wide association and interaction studies.

**Electronic supplementary material:**

The online version of this article (10.1007/s00439-019-02069-7) contains supplementary material, which is available to authorized users.

## Introduction

The study of population structure allows assigning individuals to distinct ethnic groups cohabiting a particular region (Liu et al. 2018), investigating migrations from the origin of admixed populations (Haber et al. 2016), and quantifying and characterising confounding due to shared genetic ancestry in association studies (Wang et al. 2018). In humans, genetic variation is not randomly distributed across the world because of non-random mating between individuals, who tend to marry within their community, often driven by physical proximity (Schneider and Peischl 2011). This circumstance causes variation in the relative frequency of different genotypes between groups of individuals, which may be further adapted over time through phenomena such as genetic drift or selection. Migration from a population group into a new region will lead to certain (from limited to complete) admixture between the ancestral groups resulting in a new admixed descendant population. Hence, migration will contribute to population substructure, with individuals displaying varying admixture levels of their ancestral groups (Criollo-Rayo et al. 2018).

African populations are the oldest populations in the evolution of modern human species, given its single origin in the African continent between 200,000 years ago [as indicated by mitochondrial DNA; (reviewed in Rito et al. 2013)] and 350,000 years ago (by nuclear DNA; Schlebusch et al. 2017), dates which are corroborated by archaeological evidence (Hublin et al. 2017; Richter et al. 2017). Europeans and Asians descended from a small group of Africans that migrated out-of-Africa around 60,000 years ago (see discussion around the theme of origin and date of the out-of-Africa group in (Rito et al. 2019). Africa covers some 20% of earth’s total land surface, amounting to 30 million km^2^ of diverse biomes from rainforest to woodland to savanna to desert to Mediterranean littoral environments. Africa also bears a higher amount of remaining hunter-gatherer communities than the rest of the world, from Khoisan in the southern desert, to Hadza in the Savannah, Pygmies in the tropical rainforests, and Fulani and Daza in the Sahel (Černý et al. 2011). African languages have been classified into four main linguistic families (reviewed in Campbell and Tishkoff 2010): Niger-Kordofanian spoken by agriculturalist populations across a broad geographic distribution in Africa; Afroasiatic spoken mainly by northern and eastern African pastoralists; Nilo-Saharan spoken predominantly by central and eastern African pastoralists; and the click-consonant Khoisan language spoken by eastern and southern African hunter-gatherers. These myriads of environments, climates, diets, lifestyles, and exposure to infectious diseases contribute to strong selective pressures (Campbell and Tishkoff 2008; Teo et al. 2010) upon the African populations, whose genome-wide characterization has enormous potential in revealing main aspects of human population history and genetic susceptibility to diseases.

In the reference study of African and African American genetic diversity surveyed with 1327 microsatellites, the broad continental population structure largely followed self-described ethnic and linguistic groups (Tishkoff et al. 2009). Considerable geographical extensions display remarkable homogeneity for the parts of the African continent when applying frequency model-based clustering strategies. This homogeneity is verified in the Western African populations of the Sahel Belt (Triska et al. 2015; Patin et al. 2017) when using ADMIXTURE (Alexander et al. 2009). These populations present varying proportions of only two clusters, one being more frequent in Atlantic Western populations (e.g., 90% in Mandenka), whereas the other is more frequent in Western/Central populations, especially in Esan and Yoruba of Nigeria (reaching 74–81% frequency). The main Atlantic Western component probably represents the more ancestral background of the region, while the main Western/Central component probably represents the Bantu migration initiated 5000 years ago from the Nigeria/Cameroon region. The Bantu migration further massively disrupted the original African ancestry southerly of its point of origin and affected to some extent the more southern Sahelian populations. This pattern is displayed by the various commonly used model-based clustering methods [e.g., STRUCTURE (Pritchard et al. 2000), ADMIXTURE (Alexander et al. 2009), and AWclust (Gao and Starmer 2008)] and the visual summaries of the variation in low dimensions (e.g., Principal Component Analysis (PCA) (Abegaz et al. 2018). STRUCTURE and ADMIXTURE are used to determine how individuals are inherited from a certain number of population ancestries (*K*) using maximum likelihood estimation from SNPs (Alexander et al. 2009), while PCA refers to a relatively small number of uncorrelated variables derived from an initial pool of variables while explaining as much of the total variance as possible. A higher resolution in population clustering is only obtained when fine-scale structure detection tools are applied, including haplotype-based clustering (e.g., fineSTRUCTURE jointly with CHROMOPAINTER (Lawson et al. 2012) and iterative pruning method for clustering [e.g., iNJclust (Limpiti et al. 2014), and SHIPS (Bouaziz et al. 2012), and ipPCA (Intarapanich et al. 2009)]. fineSTRUCTURE and CHROMOPAINTER have been already applied to the African context (Busby et al. 2016; Patin et al. 2017) and solved Western African clustering to a fine-scale magnitude, showing that most sub-Saharan populations share a certain proportion of ancestry with groups from outside of their current geographic region (sharing between different ethnolinguistic groups, for example, western Bantu speakers having some input from western Pygmies) as a result of gene-flow within the last 4000 years. The ipPCA method has been compared to STRUCTURE, BAPS (Corander et al. 2008), and AWclust algorithms, and outperformed these methods in achieving higher accuracy in terms of a number of obtained clusters and individual allocations to clusters in highly structured populations with closely related subpopulations (Intarapanich et al. 2009; Limpiti et al. 2011) such as in Thai population (Wangkumhang et al. 2013). However, IPCAPS was not yet applied to African populations.

We have recently implemented IPCAPS methodology, to overcome some of the shortcomings of ipPCA (Chaichoompu et al. 2017, 2019), such as restriction of a binary splitting of data into nested data sets, outlier sensitivity, and non-straightforward accommodation mixed-input data types. In this work, we applied IPCAPS to genome-wide characterized Western African samples (1396 individuals distributed over 25 ethnic groups, genotyped for 320,007 SNPs) to establish a proof-of-concept of IPCAPS as a fine-scale population substructure detection tool. IPCAPS clustering results in Western African populations were compared with ADMIXTURE and fineSTRUCTURE results. SNPs contributing to the clusters were also annotated in terms of possible functional impact, contributing information for genetic epidemiology.

## Materials and methods

### Samples

**Fig. 1:**
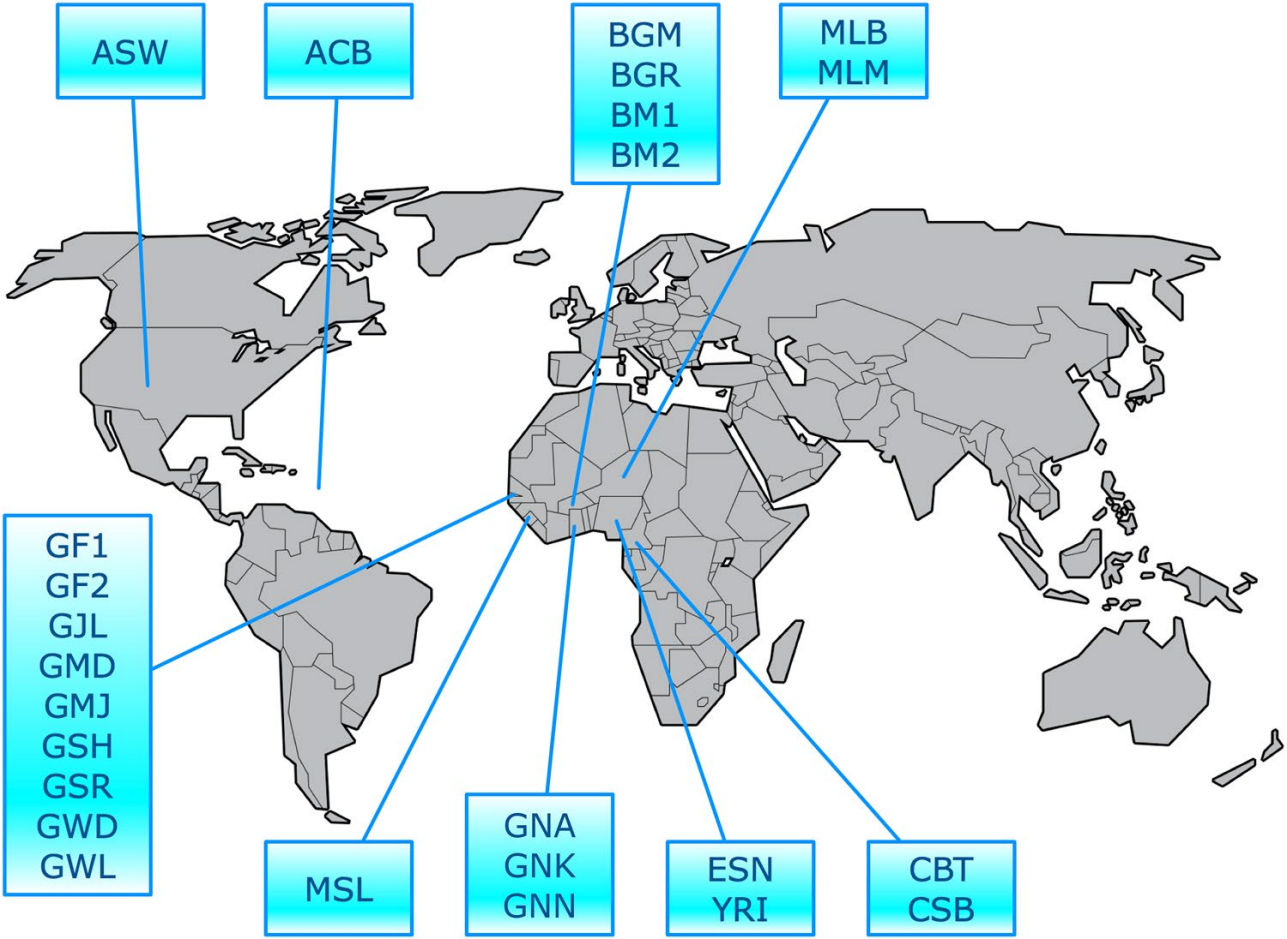
Geographical location of the African data set analyzed in this work. Abbreviations identify the following populations: *ACB* African Caribbean in Barbados, *ASW* African ancestry in Southwest USA, *BGM* Gamache in Burkina Faso, *BGR* Gurunsi in Burkina Faso, *BM1* Mossi I in Burkina Faso, *BM2* Mossi II in Burkina Faso, *CBT* Bantu in Cameroon, *CSB* Semi-Bantu in Cameroon, *ESN* Esan in Nigeria, *GF1* Fula I in Gambia, *GF2* Fula II in Gambia, *GJL* Jola in Gambia, *GMD* Mandinka II in Gambia, *GMJ* Manjago in Gambia, *GNA* Akans in Ghana, *GNK* Kasem in Ghana, *GNN* Nankam in Ghana, *GSH* Serehule in Gambia, *GSR* Serere in Gambia, *GWD* Gambian in Western Division, Mandinka, *GWL* Wollof in Gambia, *MLB* Bambara in Mali, *MLM* Malinke in Mali, *MSL* Mende in Sierra Leone, *YRI* Yoruba in Ibadan, Nigeria

We combined African genotype data from three sources (the datasets published in The 1000 Genomes Project Consortium 2012; Triska et al. 2015; Busby et al. 2016). The combined data include ACB (African Caribbean in Barbados), ASW (African ancestry in Southwest USA), BGM (Gurmatche in Burkina Faso), BGR (Gurunsi in Burkina Faso), BM1 (Mossi I in Burkina Faso), BM2 (Mossi II in Burkina Faso), CBT (Bantu in Cameroon), CSB (Semi-Bantu in Cameroon), ESN (Esan in Nigeria), GF1 (Fula I in Gambia), GF2 (Fula II in Gambia), GJL (Jola in Gambia), GMD (Mandinka II in Gambia), GMJ (Manjago in Gambia), GNA (Akans in Ghana), GNK (Kasem in Ghana), GNN (Nankam in Ghana), GSH (Serehule in Gambia), GSR (Serere in Gambia), GWD (Gambian in Western Division—Mandinka), GWL (Wollof in Gambia), MLB (Bambara in Mali), MLM (Malinke in Mali), MSL (Mende in Sierra Leone), and YRI (Yoruba in Ibadan, Nigeria). All these groups are sedentary population, except the Fulani (GF1 and GF2) who are nomadic and display high endogamy, being usually quite distinct in ADMIXTURE analyses (see for instance results in Triska et al. 2015). Samples from America (ASW) and Barbados (ACB) are also mainly of Western African ancestry. The 1000 Genomes data are from complete genome sequencing, while the other samples were screened on the Illumina Omni 2.5 M chip, although the freely available data set by Busby et al. (2016) is limited to 328,000 autosomal SNPs. For this reason, we began by checking SNPs present on Busby et al. (2016) versus the other two projects, using bcftools (Li 2011), and merging of the common SNPs to all data sets was performed using PLINK with default settings. Thus, the final data set contains 1396 individuals distributed over 25 populations, as indicated in Fig. 1 and in Supplementary Table S1 (Online Resource 1), genotyped for 320,007 SNPs after merging all data sets.

### Quality control and data preparation

Data were subjected to a rigorous quality control protocol via PLINK routines (Purcell et al. 2007). Next, we describe the steps of the adopted protocol in more detail.

Step 1: Select only founders or unrelated individuals, using the PLINK option “–filter-founders”. Non-founders are excluded before PCA-computations because they can bias the interpretation of components. In this context, founders are referred to parents, and non-founders are referred to offspring.
Step 2: Select only autosomal chromosomes 1–22, via the PLINK option “–not-chr 0,x,y,xy,mt”. This option avoids detecting structures that are gender-biased.
Step 3: Filter out SNPs in linkage disequilibrium (LD) blocks using the PLINK option “–indep-pairwise 50 5 0.2”. We assume low or no correlation structure between SNPs as suggested via *r*^2^ < 0.2, with *r*^2^ the commonly used measure of LD (Zou et al. 2010). LD pruning in this way helps to avoid that strong LD blocks drive the most important principal components or cause computational instability with classical approaches to compute PCs such as eigenvalue decomposition or EM algorithm (Raiko et al. 2008). In IPCAPS, PCs are computed by default via eigenvalue decomposition.
Step 4: Remove SNPs which the Hardy–Weinberg equilibrium (HWE) assumption is rejected, through the PLINK option “–hwe 0.001”. This step is similar to standard operating procedures in Genome-Wide Association Studies.
Step 5: Allow individuals with a call rate at least 95% by specifying “–mind 0.05” in PLINK. This step is similar to standard operating procedures in Genome-Wide Association Studies.
Step 6: Filter out missing genotypes > 2%; PLINK option “–geno 0.02”. SNPs that have a high rate of missingness should also be removed. In the presence of extensive missing data, and in particular with a missingness process that is not “missing completely at random”, biased results may be obtained. Note that the default imputation strategy within IPCAPS is single imputation by the most frequent observation (per SNP).
Step 7: Remove SNPs with a low minor allele frequency (MAF < 0.05) through the PLINK option “–maf 0.05”. Too rare SNPs (MAF < 0.05) may be found at an individual level, but not commonly presented in a population level.

All interim results of the QC analysis of 25 African populations are detailed in Supplementary Table S2 (Online Resource 1). After data QC-ing, 1396 individuals and 138,111 SNPs remained.

### Structure detection analysis strategy using IPCAPS

The IPCAPS methodology is explained in (Chaichoompu et al. 2017, 2019) and it uses PCA-based high-dimensional clustering to assign individuals to subpopulations (fine population stratification) without using assumptions of population membership or ancestry. It is available as an R package (Chaichoompu et al. 2018a). IPCAPS aims first to identify the rough or large-scale structure and second to obtain fine-scale substructure in nested data sets. The iterative analyses in PCA space come to an end via a combination of stopping criteria: a novel heuristic called EigenFit (Chaichoompu et al. 2017), mixture model-based clustering, and the average of population genetics fixation index (*F*_ST_) calculated from SNPs using Hudson’s method (Bhatia et al. 2013). Outlying individuals are separated via the RubikClust algorithm (see the R package KRIS, Chaichoompu et al. 2018b). The latter uses the concept of rotation in 3-dimensions, determined by the first three principal components (PC1, PC2, and PC3), to search for clear separation in all dimensions.

The four steps adopted in our proposed structure detection strategy using IPCAPS are as follows:

Step 1: Population clustering by IPCAPS. The PLINK binary format file (BED), obtained after the described QC protocol, was used in conjunction with IPCAPS, where the parameters were method = ‘mix’, missing = NA, covariate = NA, min.fst = 0.0008. The parameter threshold was varied in the range of 0.03–0.18, and min.in.group was varied in the range 5–20. Note that the power of the IPCAPS analysis can be improved by fine-tuning the threshold value and the min.in.group value. Details about the minimum and maximum threshold of IPCAPS can be found in (Chaichoompu et al. 2017) and is referred to therein as EigenFit criterion. The information about the country of origin and the geographical region was used only in the graphical output of IPCAPS.
Step 2: Admixture profiling. This step aims to check for the agreement between IPCAPS clusters and ADMIXTURE profiles. As in step 1, PLINK output files, after having performed QC of data, were used directly as input to the ADMIXTURE software, version 1.3.0. ADMIXTURE was run with *K* starting from 2 to 10, and the optimal number of ancestries (*K*) was obtained by tenfold cross-validation (–cv = 10). Exceptional clusters (i.e., clusters of rather outlying individuals) were ignored to visualize admixture profiling.
Step 3: Assessing identified clustering using haplotype-based analysis. As similar to Step 1, the data which have processed through the QC steps were passed as input to fineSTRUCTURE. Genotypes were phased with SHAPEIT v2.r79044 (Delaneau et al. 2012) using the 1000 Genomes phased data (The 1000 Genomes Project Consortium et al. 2015) as a reference panel and the HapMap phase 2 genetic map (The International HapMap Consortium 2007). Population structure of the phased data was evaluated using the fineSTRUCTURE v2.07 package 18 (Lawson et al. 2012) with Chromopainter v2.0 18 (Lawson et al. 2012). From the results, a dendrogram was inferred to visualize the number of statistically defined clusters that describe the data and to compare with the identified clusters from Step 1. The information about the country of origin was used only to visualize the result of fineSTRUCTURE.
Step 4: Discriminator identification. Pairwise *F*_ST_ distances were calculated for all possible pairs of clusters identified by IPCAPS (except the clusters of outliers) using the function top.discriminator. For each pair of clusters, the SNPs with high *F*_ST_ in the top percentile of 99.9% were selected for subsequent discriminant analyses. The cluster labels assigned by IPCAPS were permuted for 10,000 times to assess that a set of top-*F*_ST_ SNPs for cluster pairs was not randomly selected. In each round, the individuals were randomly resampled without replacement to reassign cluster labels as identified in step 1. Later, a *p* value was estimated from the combined set of the top-*F*_ST_ SNPs from IPCAPS groups and from resampling groups using Jaccard/Tanimoto similarity test (Chung et al. 2019) from the R package ‘jaccard’, where the parameters were method = ”bootstrap” and *B* = 1000. Among 10,000 *p* values from all iterations of each cluster pair, a maximum *p* value was used to determine the uniqueness of top-*F*_ST_ SNP set. All lists of discriminant SNPs were checked for gene annotation using the online Variant Effect Predictor tool (VEP) (McLaren et al. 2016) to assess the biological interpretation. VEP was set to enquire the transcript databases from Ensembl and GENECODE transcripts with 5000 base pairs for upstream and downstream distances.
Step 5: Functional annotation of discriminators. The list of genes obtained from VEP was checked for the functional annotations using the online tool called FUMA (Watanabe et al. 2017). Selections of discriminant genes were investigated for their enrichment of GWAS Catalog (Buniello et al. 2019) hit genes. Functional enrichment analysis was performed using FUMA across gene expression data sets from GTEx v7 with 30 general tissue types by excluding the major histocompatibility complex (MHC) region. The mapping was done for at least two overlapping genes with gene sets. For enrichment testing, *p* values were adjusted for multiple tests using Benjamini–Hochberg’s FDR control (Benjamini and Hochberg 1995).

### Identifying subgroups with similar ADMIXTURE profiles

IPCAPS groups with similar ADMIXTURE profiles were identified. Corresponding pairwise discriminator genes were functionally annotated, as in Step 5 above, to highlight functionally relevant differences between the groups and thus to seek evidence for them to constitute two groups.

## Results

### Overview of samples

**Fig. 2:**
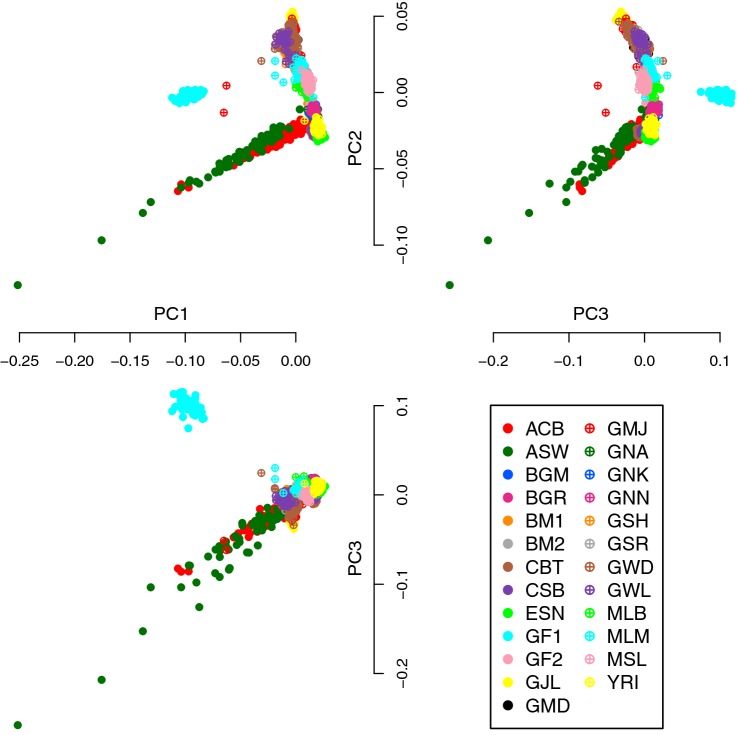
The first three principal components of the entire African data set before IPCAPS clustering. Highlighted points refer to ethnic groups

**Fig. 3:**
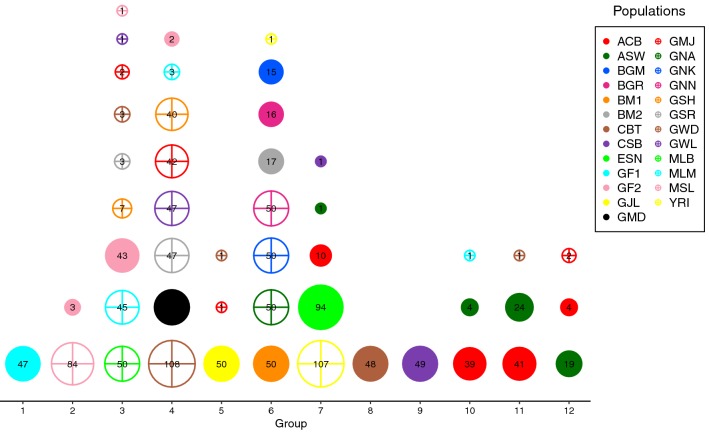
Bubble plot of the IPCAPS clusters that shows the distribution of how cluster members are composed

After passing through all quality control steps, the number of SNPs was reduced from 320,007 to 138,111; no individual was removed (i.e., 1396 individuals contributed to the subsequent analyses). The intermediate results of all quality control steps are shown in Supplementary Table S2. After having submitted the QC-ed data to PCA, it was difficult to identify clear differences between the 25 input populations (Fig. 2). Only GF1 individuals (Figs. 2, 3, solid cyan circle) were well separated from the other populations, which are in line with the large genetic distance of GF1 from the other African populations (*F*_ST_ ≥ 0.14, Supplementary Table S3 in Online Resource 1). Although this ethnic group is an entirely distinct nomadic group, it is curious that they split off from a second Fulani group from the same country. We can hypothesize that this last Fulani group GF2 may have had higher gene flow with the neighbors of sedentary groups, being thus genetically more similar. We furthermore observed that ACB (solid red circle) and ASW (solid green circle) were spread out, unlike the other populations (Figs. 2, 3). Notably, these two groups are African descendants living in the American continent (in Barbados and the southwest USA, respectively), and, as such, are descendants from enslaved Africans that originated from diverse parts of Western Africa. It is known that there is still 10% European admixture in African Americans (Patin et al. 2017), which may explain the differences observed in these two groups together with the Western African groups.

### Fine-scale structure detection of Western Africans via IPCAPS

**Fig. 4:**
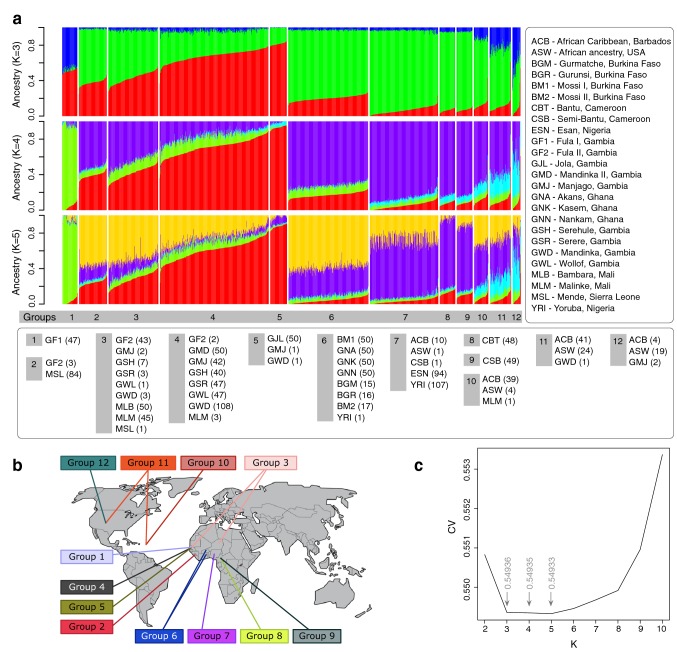
**a** ADMIXTURE clustering of the African data set. The numbers of ancestry groups (*K*) are between 3 and 5. The numbers (1–12) under the ADMIXTURE plot represent the IPCAPS groups. The group members are listed underneath the plot; the numbers in parentheses represent the numbers of individuals from those ethnic groups. **b** Geographic map showing, for each group, the geographic origin for the majority (less than five individuals) of group members. **c** Cross-validation (cv) error from ADMIXTURE based on tenfold cross-validation

To avoid too high dispersion compared to subgroups imposed by reference labels, the IPCAPS’s result run on the QC-ed data with threshold = 0.18 and min.in.group = 20 was selected to explain in this section. IPCAPS analysis revealed 12 groups instead of the initial 25 self-identified population groups (Supplementary Table S4 in Online Resource 1). In addition, 22 individuals were separated into 9 groups with less than 5 individuals per group; these individuals were considered to be outliers and not considered for subsequent analyses. For comparisons purposes, the ADMIXTURE profiling plots for IPCAPS groups 1–12 are shown in Fig. 4a, for optimal *K* ancestors 3–5 (cross-validation error in Fig. 4c). In Fig. 4b, the geographic map shows the geographic origin for the majority (less than five individuals) of group members for each group.

IPCAPS group 1 (Figs. 3, 4a) is composed of one self-identified population group, as expected the nomadic Fulani GF1 which was also individualized in ADMIXTURE, along all *K*s. IPCAPS groups 2, 3, 4 and 5 individualize individuals that in ADMIXTURE *K* = 5 form quite a continuum in the alternative proportions between two components represented in yellow and in red. These individuals belong to populations from the westernmost countries; the Gambia and Sierra Leone, and neighboring Mali. IPCAPS is almost able to isolate Mende from Sierra Leone (group 2) and Jola from Gambia (group 5), which somewhat show also an extreme position in the yellow–red spectrum in ADMIXTURE, while group 3 gets most Mali (Bambara and Malinke) and the other Fulani group individuals mixed with a few Gambian, in contrast with group 4 made of most of all other Gambian individuals (Mandinka, Manjago, Serehule, Serere, and Wollof). IPCAPS groups 6, 7, 8 and 9 present a distinctive ADMIXTURE *K* = 5 particularity of higher amount of the ancestry represented by the violet color mixed with variable proportions of the already mentioned yellow and red components. IPCAPS group 6 is composed of Burkina Faso (Gurmatche, Gurunsi, and Mossi) and neighboring Ghana (Akans, Kasem, and Nankam) population groups, which display a quite homogeneous pattern in ADMIXTURE. IPCAPS group 7 is made of the Nigerian groups (Esan and Yoruba) and some African Caribbean. IPCAPS groups 8 and 9 are made of the two Cameroon populations, which are of Bantu origin (full Bantu in 8 and semi-Bantu in 9) who were not distinguishable in ADMIXTURE *K* = 5. IPCAPS groups 10, 11, and 12 are mostly African Americans from USA and Barbados that in ADMIXTURE present variable proportions of the cyan color reflecting Caucasian admixture.

**Fig. 5:**
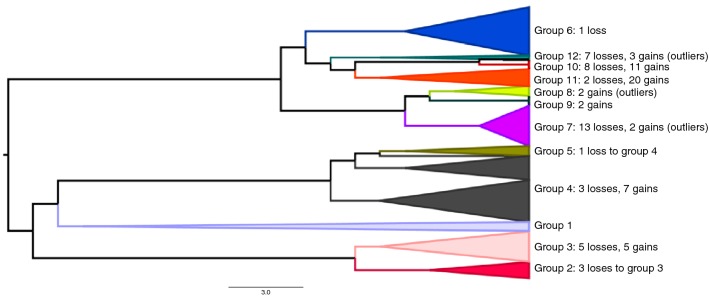
Concordance analysis between IPCAPS and fineSTRUCTURE. The dendrogram represents the identified groups by fineSTRUCTURE. These groups were uniquely matched to the 12 groups identified by IPCAPS; differences between the matched groups are indicated taking IPCAPS groups as reference

We further ascertained the potential of the haplotype-based fineSTRUCTURE method in solving population structure in the tested data set. When applied directly in the pruned data set (to be directly compared with our results), fineSTRUCTURE identified 29 groups (at least 5 individuals per group; some individuals were in long individual branches, as shown in Fig. 5). Superimposing the 12 IPCAPS groups onto the fineSTRUCTURE dendrogram (Fig. 5) by manual matching, a satisfactory agreement was observed, except for group 4. Of the 1374 individuals that were allocated to the same IPCAPS group, only 43 of them (3%) would not be allocated into the same fineSTRUCTURE groups. A more traditional fineSTRUCTURE analysis (unpruned data set) revealed 38 groups at the tip level (with at least five individuals per group; not shown). This fineSTRUCTURE, at its maximum, did not solve all ethnic group affiliations, especially within and between Gambian and Mali groups.

### Annotation of discriminator diversity

The pairwise discriminators were selected from the corresponding 99.9th percentile of SNP-wise *F*_ST_ derived per pairwise IPCAPS group comparison. The average number of top-*F*_ST_ SNPs thus identified across pairs of groups is 138.79. The minimum number of top-*F*_ST_ SNPs is 134 SNPs, and the maximum number of top-*F*_ST_ SNPs is 139. All SNPs were mapped to genes using VEP (details provided in Online Resource 2). For the genes mapped to these 66 top-F_ST_ SNP lists (number of combinations of 2 IPCAPS groups that can be selected from 12 groups), we used FUMA to perform gene enrichment analysis. In particular, we linked the query genes (2580 genes in total), corresponding to the pooled genes of 66 top-*F*_ST_ SNP lists, to the GWAS Catalog. This led to a total of 489 GWAS Catalog genes, significantly linking to several phenotypes including response to fenofibrate, response to chemotherapy in breast cancer hypertensive cases, obesity-related traits, body mass index, post-bronchodilator FEV1/FVC ratio, amyotrophic lateral sclerosis, post-bronchodilator FEV1, night sleep phenotypes, height, asthma, etc. (Table 1). Table 2 displays the top two entries of Table 1, for which the hit genes from the GWAS Catalog were also among the 2580 genes, as described before.

**Table 1:** The top 30 of GWAS traits according to adjusted p-values

GWAS catalog trait	Total Nr of genes*	Total Nr of matching genes**	Multiple testing adjusted, *p* value
Response to Fenofibrate	3	3	0
Response to chemotherapy in breast cancer hypertensive cases (cumulative dose) (Bevacizumab)	6	6	0
Obesity-related traits	756	168	6.40E−59
Body mass index	546	109	3.00E−33
Post-bronchodilator FEV1/FVC ratio	199	60	1.73E−28
Amyotrophic lateral sclerosis (sporadic)	164	46	2.09E−20
Post-bronchodilator FEV1	120	36	3.90E−17
Night sleep phenotypes	538	81	1.20E−16
Height	522	78	7.10E−16
Asthma	207	45	2.41E−15
Myopia	73	25	1.02E−13
Diisocyanate-induced asthma	189	39	1.40E−12
Post-bronchodilator FEV1/FVC ratio in COPD	51	20	1.65E−12
Alzheimer’s disease (cognitive decline)	48	19	4.91E−12
Menarche (age at onset)	216	41	5.42E−12
Blood pressure (smoking interaction)	29	15	5.59E−12
Type 2 diabetes	255	45	5.60E−12
Coronary artery disease	431	61	1.40E−11
Schizophrenia	604	75	3.03E−11
Photic sneeze reflex	65	21	3.03E−11
Response to amphetamines	33	15	6.02E−11
Plateletcrit	219	39	1.19E−10
Autism spectrum disorder, attention deficit-hyperactivity disorder, bipolar disorder, major depressive disorder, and schizophrenia (combined)	46	17	1.98E−10
Lung adenocarcinoma	113	26	6.70E−10
Systemic lupus erythematosus	222	38	6.81E−10
Platelet count	285	44	8.07E−10
Major depressive disorder	133	28	1.18E−09
Coronary artery calcified atherosclerotic plaque (130 HU threshold) in type 2 diabetes	35	14	2.15E−09
Body mass index (joint analysis main effects and smoking interaction)	81	21	2.90E−09
Intraocular pressure	82	21	3.65E−09

The adjusted *p* values determine the significance level from gene-set enrichment testing of matched genes and the reported genes in GWAS Catalog. The *p* values were adjusted using the method of Benjamini–Hochberg (FDR)

*From the GWAS Catalog

**From IPCAPS, derived from 66 top-*F*_ST_ SNP lists, as described in the text

**Table 2:** The information of the first two gene sets which have the exact match for all reported genes in GWAS Catalog (*p* value = 0)

GWAS catalog trait	Gene	Chr	SNP	Location on gene	Cluster pair
Response to Fenofibrate	CD36	7	rs10246082	Intron	2–7
			rs7779873	Intron	2–7, 3–7, 4–7, 5–7, 7–9
			rs3211881	Intron	8–10
	DOCK4	7	rs2729536	Intron	1–8, 1–11, 1–12, 8–9
			rs6951506	Intron	2–10, 8–10
	NXPH1	7	rs7812117	Intron	3–4, 4–8, 8–10
			rs6978212	Intron	3–5, 5–6, 5–7, 5–8
			rs6955389	Intron	4–11
Response to chemotherapy in breast cancer hypertensive cases (cumulative dose) (Bevacizumab)	MAML2	11	rs514686	Intron	1–2
			rs10501841	Intron	2–7
			rs7104859	Intron	3–5, 5–6, 5–7
			rs7951485	Intron	6–7
			rs555329	Intron	9–10
	MSRA	8	rs11250004	Intron	1–2, 1–9
			rs11775334	Intron	2–3, 2–4, 2–6
			rs11993663	Intron	3–9
	PARVB	22	rs5764495	Intron	9–12
	PFKFB3	10	rs2516614	Intron	2–6
	SV2C	5	rs12522470	Intron	2–3
			rs2081076	Intron	11–12
	TGFBR2	3	rs9881945	Intron	2–7

The cluster pairs are the pairwise comparison for all possibilities of detected groups by IPCAPS

**Fig. 6:**
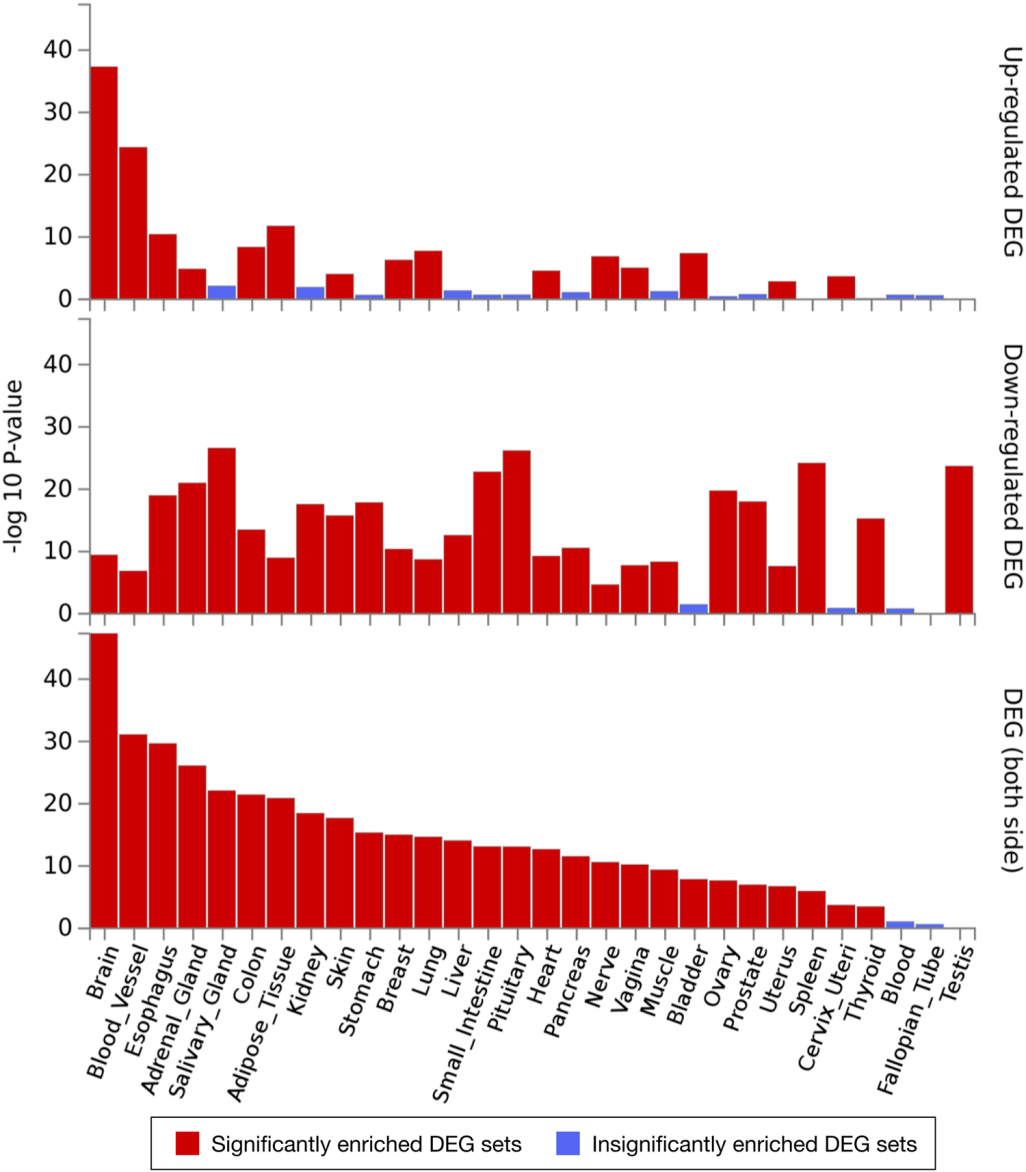
Tissue specificity related to the differentially expressed genes (DEG) derived from the top-*F*_ST_ SNPs (99.9th percentile) across all cluster comparisons. A distinction is made between upregulated DEG (top), downregulated DEG (middle), and bidirectional DEG (bottom). The *p* values represent the probability from the hypergeometric test

The GWAS-obtained gene set for the response to fenofibrate consists of three genes, *CD36*, *DOCK4*, and *NXPH1*, from chromosome 7. Fenofibrate is medicine for lowering high cholesterol and triglyceride levels. All discriminative SNPs linked to these three genes are located in introns: rs10246082, rs7779873 and rs3211881 for *CD36*; rs2729536 and rs6951506 for *DOCK4*; rs7812117, rs6978212, and rs6955389 for *NXPH1*. The GWAS set of genes associated with response to chemotherapy in breast cancer hypertensive cases in cumulative dose consists of six genes. This chemotherapy refers to Bevacizumab, which is used to treat colorectal, lung, glioblastoma, kidney, cervical, and ovarian cancer. These six genes, *MAML2*, *MSRA*, *PARVB*, *PFKFB3*, *SV2C*, and *TGFBR2*, bear, respectively, the following intronic discriminative SNPs: rs514686, rs10501841, rs7104859, rs7951485, and rs555329; rs11250004, rs11775334, and rs11993663; rs5764495; rs2516614; rs12522470 and rs2081076; and rs9881945. Furthermore, the 2580 query genes were widely regulated in several tissues, including brain, blood vessel, esophagus, adrenal gland, salivary gland, colon, adipose tissue, kidney, skin, stomach, breast, lung, liver, small intestine, pituitary, heart, pancreas, nerve, vagina, muscle, bladder, ovary, prostate, uterus, spleen, cervix uteri, thyroid, and testis (Fig. 6).

### Annotation of discriminators in the IPCAPS groups with highly similar ADMIXTURE profiles

**Fig. 7:**
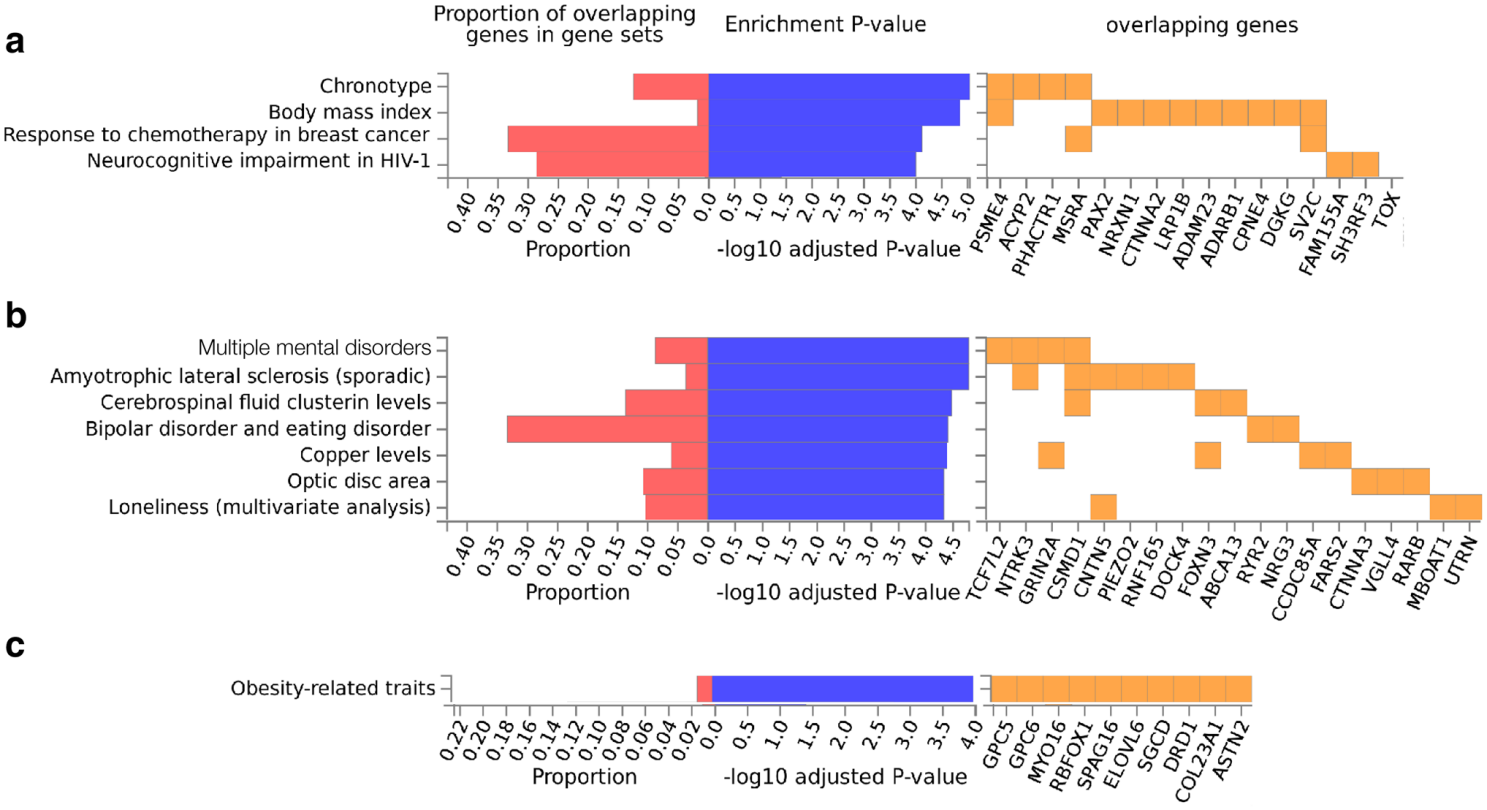
The lists of genes that are associated with the top-*F*_ST_ SNPs (99.9th percentile) between groups 2 and 3, groups 8 and 9, and groups 10 and 11, is shown in **a, b**, and **c**, respectively, obtained from FUMA. The listed genes (orange) from genome-wide association studies obtained from the GWAS Catalog (Buniello et al. 2019). The proportions of overlapping genes in gene sets are shown in red, and the enrichment *p* values are shown in blue. The lists of GWAS experiments were filtered by enrichment *p* value ≥ 0.0001 (− log_10_(0.0001) = 4)

IPCAPS groups 2 and 3 have similar ADMIXTURE profiles (Fig. 4a), which is also the case for groups 8 and 9, and for groups 10 and 11. From permutation, the maximum *p* value of the discriminant SNPs between IPCAPS groups 2 and 3 is 0.001. Enrichment analysis performed on the discriminator genes between group 2 (mostly from Sierra Leone) and group 3 (mostly from Mali and Gambia) shows that top significant enrichments for GWAS hit genes (*p* value < 0.0001 or − log_10_(0.0001) = 4) are obtained for four GWAS traits (Fig. 7a; Supplementary Fig. S5). These include chronotype (*PSME4*, *ACYP2*, *PHACTR1*, and *MSRA*), body mass index (*PSME4*, *PAX2*, *NRXN1*, *CTNNA2*, *LRP1B*, *ADAM23*, *ADARB1*, *CPNE4*, *DGKG*, and *SV2C*), response to chemotherapy in breast cancer hypertensive cases (*MSRA* and *SV2C*), and neurocognitive impairment in HIV-1 infection (*FAM155A*, *SH3RF3* and *TOX*). Moreover, the top-F_ST_ genes for groups 2 and 3 are significantly upregulated in the blood vessel and brain, and significantly downregulated in the stomach. When considering both down- and upregulation, the genes are significantly regulated in the stomach, blood vessel, and muscle (Supplementary Figs. S6, S7, red, FUMA results).

Similarly, in the case of group 8 (CBT) and group 9 (CSB), both from Cameroon, top enrichment is obtained by FUMA for seven GWAS traits (Fig. 7b; Supplementary Fig. S8) based on the selected top-*F*_ST_ SNPs (the maximum *p* value from permutation is 0.001). These include multiple mental disorders, i.e., autism spectrum disorder, attention deficit-hyperactivity disorder (ADHD), major depressive disorder, and schizophrenia (*TCF7L2*, *NTRK3*, *GRIN2A* and *CSMD1*), amyotrophic lateral sclerosis (*NTRK3*, *CSMD1*, *CNTN5*, *PIEZO2*, *RNF165* and *DOCK4*), cerebrospinal fluid clusterin levels (*CSMD1*, *FOXN3* and *ABCA13*), bipolar disorder and eating disorder (*RYR2* and *NRG3*), copper levels (*GRIN2A*, *FOXN3*, *CCDC85A* and *FARS2*), optic disc area (*CTNNA3*, *VGLL4* and *RARB*), and loneliness (*CNTN5*, *MBOAT1* and *UTRN*). These top-*F*_ST_ genes discriminating groups 8 and 9 are significantly upregulated in the brain, blood vessel, and breast. A large set of genes is significantly downregulated in the small intestine, stomach, salivary gland, skin, spleen, esophagus, colon, testis, pituitary, thyroid, prostate, and adrenal gland. For both upregulation and downregulation, the genes are significantly regulated in the brain, colon, esophagus, stomach, blood vessel, salivary gland, breast, thyroid, vagina, small intestine, nerve, adipose tissue and liver (Supplementary Fig. S9, red). The significantly expressed genes are shown in the heat map with different levels of gene expression ranging from high (dark red) to low (dark blue), as before (Supplementary Fig. S10).

Lastly, in the case of group 10 (the majorities are ACB) compared to group 11 (mixed between ACB and ASW), corresponding discriminating genes are enriched for GWAS hits (obtained from the discriminatory SNPs with the maximum *p* value is 0.001) linked to obesity-related traits (*GPC5*, *GPC6*, *MYO16*, *RBFOX1*, *SPAG16*, *ELOVL6*, *SGCD*, *DRD1*, *COL23A1*, and *ASTN2*) as shown in Fig. 7c and Supplementary Fig. S11. In Supplementary Fig. S12 (red), the top-F_ST_ genes of groups 10 and 11 are significantly downregulated in the prostate, thyroid, salivary gland, pituitary, and esophagus, and significantly regulated in both sides for the brain, adipose tissue, and breast. Moreover, the significantly expressed genes are highlighted in a heatmap provided as Supplementary Fig. S13.

## Discussion

The rich genetic structure of Africans (Zeiger et al. 2018) has received much attention. As indicated before, large-scale migration events that occurred throughout history and a large mixture of ancestries caused a massive and subdivided population structure in Africa (Tishkoff et al. 2009). Africans have been studied from different angles, including evolutionary history (Lambert and Tishkoff 2009), prehistoric time (Skoglund et al. 2017), and migration (Schlebusch and Jakobsson 2018). Our study looks into deeper details to identify fine-scale population structure in Western African populations.

Initially, we developed IPCAPS as a methodology to detect fine-scale structure in patients, after having removed confounding population structure. A validation of IPCAPS on real-life data for disease subtyping is difficult since there is no clear explanation of how disease subtypes should be. However, a lot of information is available about genetic substructure in general populations, and this motivated the current work. In particular, IPCAPS was built on ipPCA (Intarapanich et al. 2009; Limpiti et al. 2011), which was able to detect fine-scale structure in the Thai population (Wangkumhang et al. 2013). We adopted five major steps for general population structure detection analysis using IPCAPS. These steps involve population clustering analysis with IPCAPS, followed by admixture profiling and subgroup discriminator identification to aid in the interpretation of IPCAPS findings. IPCAPS was compared to ipPCA, and it outperformed ipPCA in all considered simulation scenarios (Chaichoompu et al. 2017). IPCAPS has the potential for detecting fine population structure using SNPs in populations, without the need for inferring haplotypes or haplotype phasing.

Among the 12 groups that were revealed out of the Western African samples, IPCAPS was able to distinguish between most countries who were not identifiable in ADMIXTURE. IPCAPS was not able to distinguish between most self-identified ethnic groups within countries which may be explained by an extensive gene flow existing between groups, a fact further supported by the many examples of individuals from one group/country that would be molecularly affiliated in another group. According to gene-set enrichment analysis, the genetic differences among these revealed groups are associated with up to 489 gene sets from GWAS, related to obesity, BMI, response to drugs (cholesterol level and chemotherapy) and cancer. This observation testifies that the genetic diversity that discriminates between these groups is of functional impact. For instance, in *CD36* gene, which responds to Fenofibrate drug used to decrease cholesterol level, two intronic SNPs (rs10246082 and rs7779873) discriminate individuals of group 7 (mainly from Nigeria) from the groups 2, 3, 4, 5, and 9 (mainly from Cameroon, Gambia, Mali, and Sierra Leone).

Even closely related groups were discriminated by IPCAPS and through functionally important discriminatory SNPs. Groups 2 (Mende from Sierra Leone) and 3 (Bambara and Malinke from Mali and Fula II from the Gambia) are discriminated by SNPs associated with chronotype (behavior according to the biological clock), BMI, response to chemotherapy in breast cancer and neurocognitive impairment in HIV-1 (enrichment *p* value < 0.0001). In particular, intronic rs4113420 SNP in *FAM155A* gene, associated with neurocognitive impairment in HIV-1, distinguishes group 2 from 3, and group 2 from other eight groups (groups 4, 5, 6, 7, 8, 9, 10 and 11). The two ethnic groups from Cameroon, Bantu (CBT) and Semi-Bantu (CSB), or groups 8 and 9, respectively, were distinguishable by SNPs related to several traits, as several mental disorders, amyotrophic lateral sclerosis, copper levels, and optic disc area. *NTRK3* gene is compelling in the multiple mental disorders, and its rs16941321 SNP is only discriminating between these two ethnic groups from Cameroon.

The fine-scale resolution on Western African ancestry structure further allowed discrimination between the African migrants to North America. ACB from Barbados and ASB from Southwest USA have a similar genetic profile to Nigerian (ESN and YRI), except for the mixture with European ancestors, contributing to differences between groups 7 and 11 (rs4886414 and rs7142344 SNPs have contrast frequencies in African and Europeans, respectively, 0.0877 and 0.7336, and 0.9251 and 0.0974). Groups 10 and 11 have similar ADMIXTURE profiles, but with a slightly different ratio of European ancestral part, with discriminatory SNPs linked to genes that enrich for obesity (enrichment *p* value < 0.0001), as *DRD1* (SNP rs686) and *COL23A1* (rs17648108—which display the lowest allele frequency in Europeans, of 0.2684, contrasting to higher than 0.5 in other population groups).

Detecting population structure can be observed at different granularity levels. The structure detection tool fineSTRUCTURE (Lawson et al. 2012) to infer fine-scale genetic substructure in populations is a Bayesian clustering method that uses sufficient statistics as input, which are in turn output from CHROMOPAINTER (Lawson et al. 2012). The latter finds haplotypes in sequence data and “paints” every individual as a combination of all other sequences. We verified that even these methods could not solve all ethnic group affiliations in agreement with self-identification. The number of inferred groups can be played around by reducing the IPCAPS threshold from 0.18 (maximum allowable threshold value) to 0.03 (minimum allowable threshold value), thus changing from 12 but 19 groups, as shown in Supplementary Fig. S14. The optimal threshold will depend on the data application and context. Regardless, haplotype-based analyses, as required for fineSTRUCTURE, are computationally intensive and time-consuming. IPCAPS avoids this and can easily be run on a personal computer, whereas finding refined and meaningful genetic substructure. For the African data set analyzed in this work, IPCAPS took less than 2 h to process on a personal computer (running in a single thread on the 3.5-GHz CPU with 16 GB of RAM). However, the haplotype-based analysis (pruned data set) took about 30 h on a cluster computer with the 2.5-GHz CPUs and 250 GB of RAM (phasing haplotype took about 4 h running in 32 threads using SHAPEIT, and total fineSTRUCTURE clustering took about 26 h, taking advantage of running CHROMOPAINTER in 64 threads).

The finding of discriminant markers among the subpopulations explained above is beneficial to raise a concern in study design for GWAS. Using country or boundary to label samples may be biased. In case of, for example, CBT and CSB (both groups are Cameroon), the GWAS experimental design for mental disorders should be well concerned since some discriminatory SNPs among these ethnic groups are associated to several mental disorders. The GWAS result is likely to be false positive when the numbers of cases and controls is not equally distributed (Bush and Moore 2012). On the one hand, unwanted signals or subpopulations should be removed before GWAS (Abegaz et al. 2018), on the other hand, subpopulations should be first detected (for instance, via IPCAPS and fineSTRUCTURE) and GWAS can be then performed on subgroups.

## Conclusion

In this work, we have reported fine-scale population structure in the Western African populations using IPCAPS as genetic clustering tool. IPCAPS (with threshold = 0.18) provides an intermediate sub-clustering resolution (12 IPCAPS groups) between ADMIXTURE (frequency-based, *K* = 5) and fineSTRUCTURE (haplotype-based, 29 groups) profiling. The number of IPCAPS groups is substantially smaller than the actual number of African ethnic groups/populations taken as input, implying that some IPCAPS groups consist of subsets of several African subpopulations. However, three meaningful fine-scale population structures were highlighted in the African populations living in Cameroon, Gambia, Mali, Southwest USA, and Barbados. As the detected hidden substructure in terms of discriminant genes could be potentially linked to several (disease) traits via their enrichment for established GWAS hits, we furthermore believe that our ability to detect such fine-scale structure in populations can also contribute to the improvement of genome-wide association studies for complex human traits.

## Electronic supplementary material

Below is the link to the electronic supplementary material.

**Supplementary Fig. S1** The first three Principal Components of the African data set after having removed outliers (the group that has less five individuals). IPCAPS revealed 12 clusters of African samples. Each such cluster is color-coded. (EPS 476 kb)

**Supplementary Fig. S2** The first three Principal Components calculated from the individuals in groups 2 and 3. (EPS 329 kb)

**Supplementary Fig. S3** The first three Principal Components calculated from the individuals in groups 8 and 9. (EPS 318 kb)

**Supplementary Fig. S4** The first three Principal Components calculated from the individuals in groups 10 and 11. (EPS 314 kb)

**Supplementary Fig. S5** The complete list of genes that are associated with the top-F_ST_ SNPs (99.9^th^ percentile of F_ST_) between groups 2 and 3, obtained from FUMA. The listed genes (orange) from genome-wide association studies obtained from the GWAS Catalog. The proportions of overlapping genes in gene sets are shown in red, and the enrichment p-values are shown in blue. (EPS 3070 kb)

**Supplementary Fig. S6** Tissue specificity related to the differentially expressed genes (DEG) derived from the top-F_ST_ SNPs between groups 2 and 3. A distinction is made between upregulated DEG (top), downregulated DEG (middle), and bidirectional DEG (bottom). The p-values represent the probability from the hypergeometric test. (EPS 1655 kb)

**Supplementary Fig. S7** A heat map of the average of log_2_ transformed expression value per tissue type between groups 2 and 3. Dark red corresponds to high gene expression versus dark blue referring to low gene expression color. (PDF 298 kb)

**Supplementary Fig. S8** The complete list of genes that are associated with the top-F_ST_ SNPs (99.9^th^ percentile of F_ST_) between groups 8 and 9, obtained from FUMA. The listed genes (orange) from genome-wide association studies obtained from the GWAS Catalog. The proportions of overlapping genes in gene sets are shown in red, and the enrichment p-values are shown in blue. (EPS 3918 kb)

**Supplementary Fig. S9** Tissue specificity related to the differentially expressed genes (DEG) derived from the top-F_ST_ SNPs between groups 8 and 9. A distinction is made between up-regulated DEG (top), down-regulated DEG (middle), and bidirectional DEG (bottom). The p-values represent the probability from the hypergeometric test. (EPS 2112 kb)

**Supplementary Fig. S10** A heat map of the average of log_2_ transformed expression value per tissue type between groups 8 and 9. Dark red corresponds to high gene expression versus dark blue referring to low gene expression color. (PDF 288 kb)

**Supplementary Fig. S11** The complete list of genes that are associated with the top discriminative SNPs (99.9^th^ percentile of F_ST_) between groups 10 and 11, obtained from FUMA. The listed genes (orange) from genome-wide association studies obtained from the GWAS Catalog. The proportions of overlapping genes in gene sets are shown in red, and the enrichment p-values are shown in blue. (EPS 2089 kb)

**Supplementary Fig. S12** Tissue specificity related to the differentially expressed genes (DEG) derived from the top-F_ST_ SNPs between groups 10 and 11. A distinction is made between upregulated DEG (top), downregulated DEG (middle), and bidirectional DEG (bottom). The p-values represent the probability from the hypergeometric test. (EPS 1679 kb)

**Supplementary Fig. S13** A heat map of the average of log_2_ transformed expression value per tissue type between groups 10 and 11. Dark red corresponds to high gene expression versus dark blue referring to low gene expression color. (PDF 256 kb)

**Supplementary Fig. S14** ADMIXTURE clustering of the African data set. The numbers of ancestry groups (K) are between 3 and 5. The numbers (1–19) in the ADMIXTURE plot represent the IPCAPS groups. The groups were obtained from IPCAPS with the threshold parameter set to 0.03. The group members are listed under the plot and the numbers in parentheses represent the numbers of individuals from those ethnic groups. (EPS 1960 kb)

Supplementary material 15 (DOCX 40 kb)

Supplementary material 16 (XLSX 1239 kb)
