# Supplementary figures and images for: A different view on fine-scale population structure in Western African populations
Source: Hum Genet. 2019 Oct 19;139(1):45–59. doi: 10.1007/s00439-019-02069-7 (PMC6942040; doi:10.1007/s00439-019-02069-7)

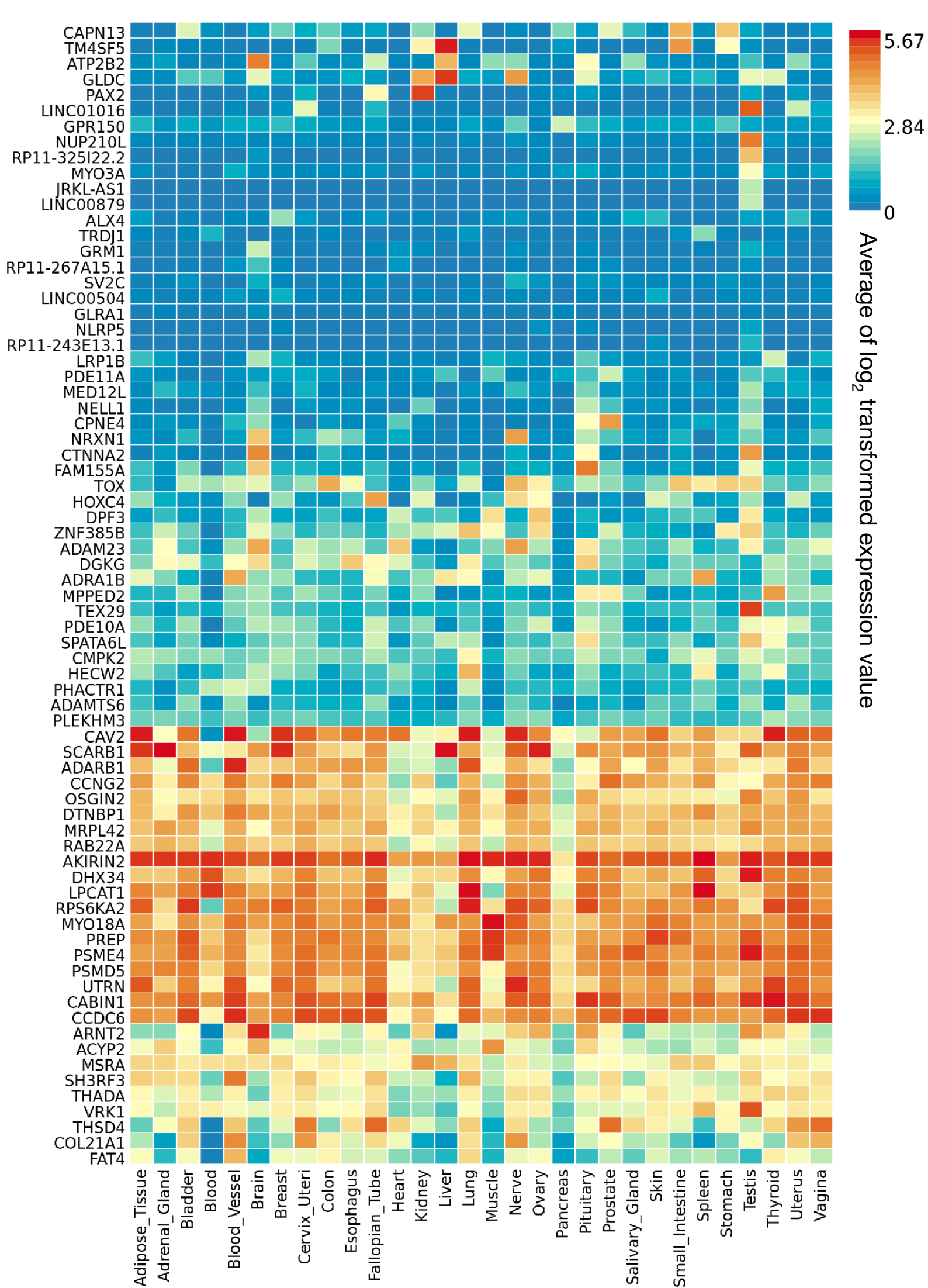

Supplement: Supplementary file 7 — Supplementary Fig. S7 A heat map of the average of log2 transformed expression value per tissue type between groups 2 and 3. Dark red corresponds to high gene expression versus dark blue referring to low gene expression color. (PDF 298 kb) [file 439_2019_2069_MOESM7_ESM.pdf]

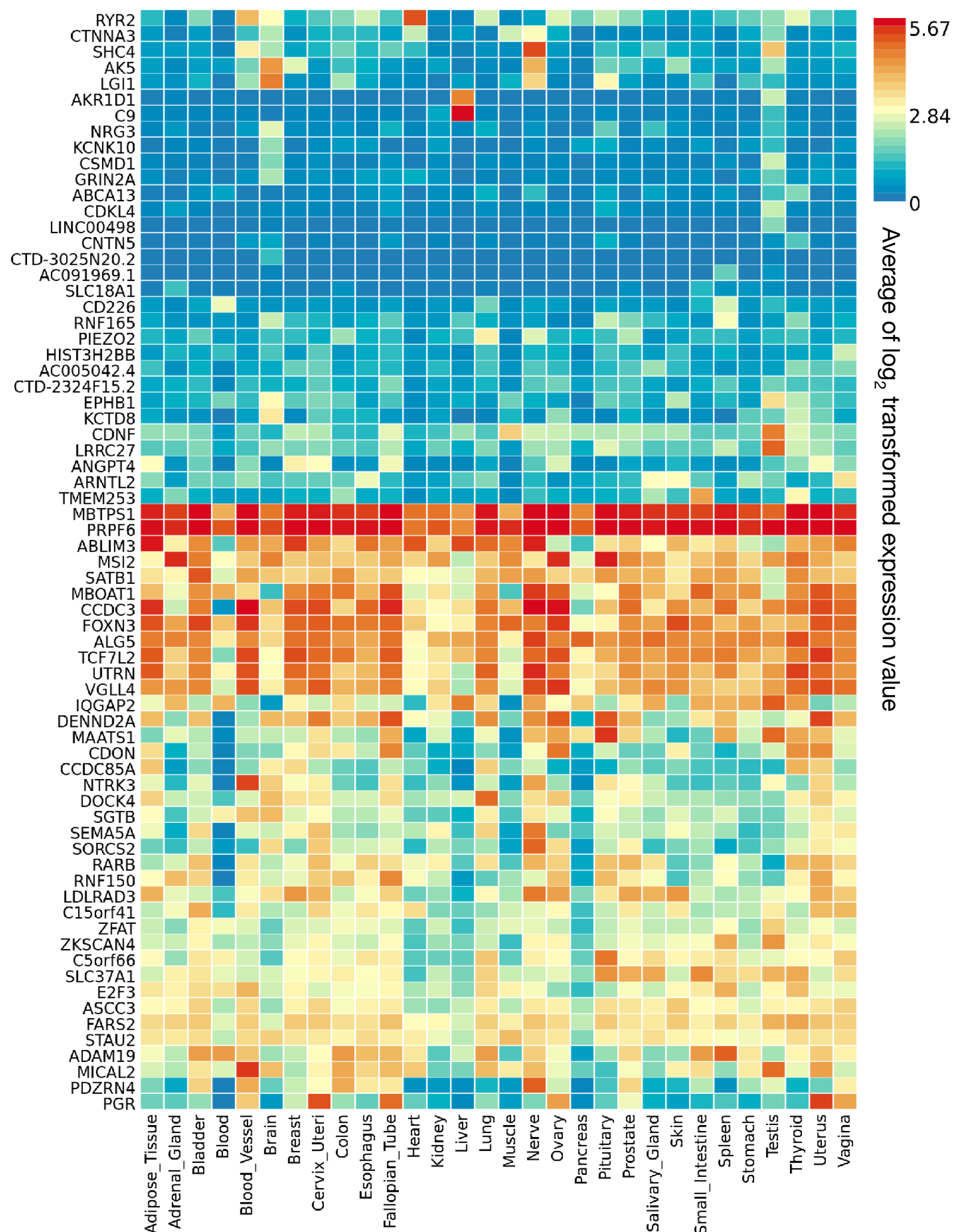

Supplement: Supplementary file 10 — Supplementary Fig. S10 A heat map of the average of log2 transformed expression value per tissue type between groups 8 and 9. Dark red corresponds to high gene expression versus dark blue referring to low gene expression color. (PDF 288 kb) [file 439_2019_2069_MOESM10_ESM.pdf]

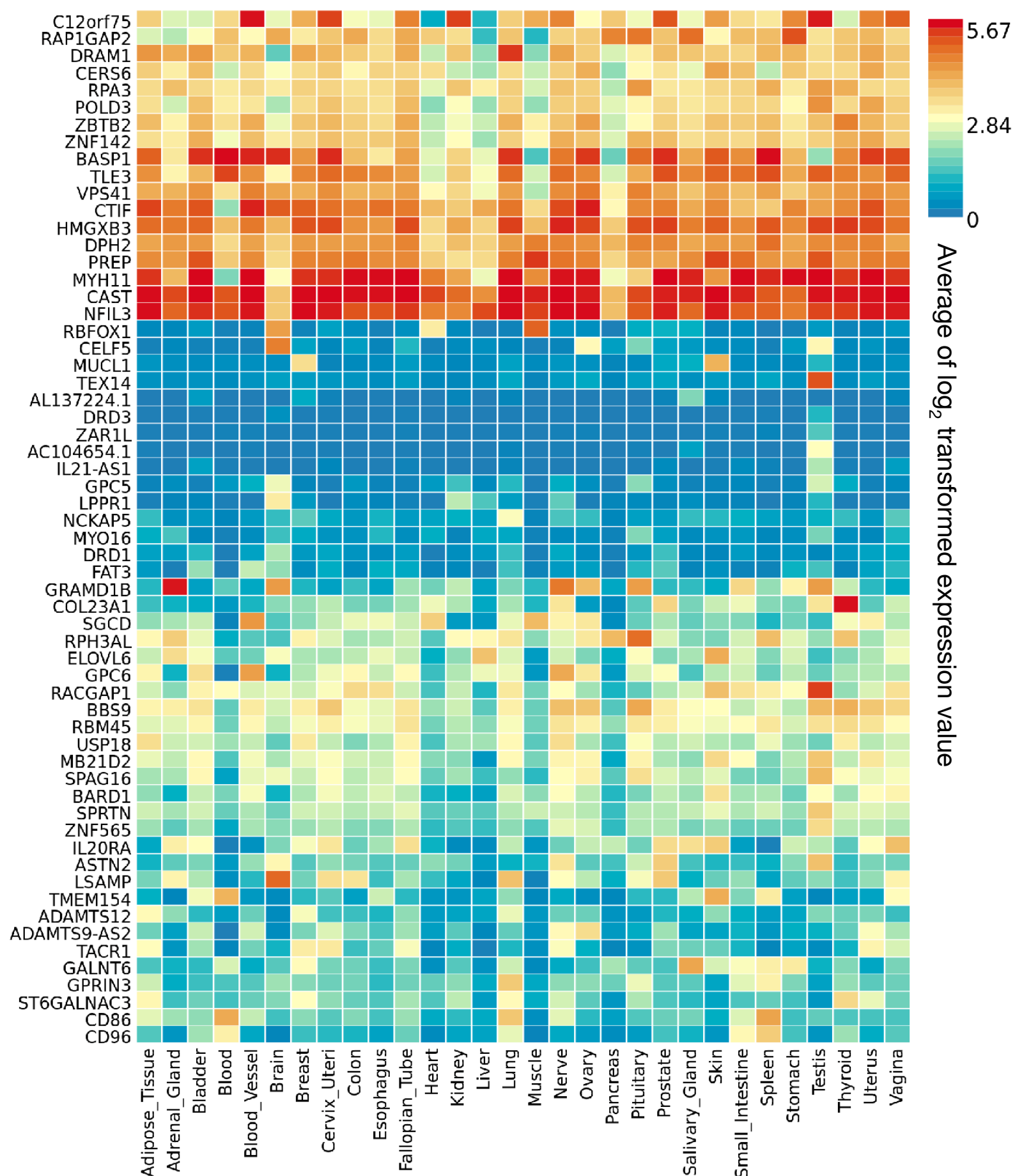

Supplement: Supplementary file 13 — Supplementary Fig. S13 A heat map of the average of log2 transformed expression value per tissue type between groups 10 and 11. Dark red corresponds to high gene expression versus dark blue referring to low gene expression color. (PDF 256 kb) [file 439_2019_2069_MOESM13_ESM.pdf]
