# Supplementary material for: A different view on fine-scale population structure in Western African populations
Source: Hum Genet. 2019 Oct 19;139(1):45–59. doi: 10.1007/s00439-019-02069-7 (PMC6942040; doi:10.1007/s00439-019-02069-7)
Supplement: Supplementary file 15 — Supplementary material 15 (DOCX 40 kb) [file 439_2019_2069_MOESM15_ESM.docx]

**Supplementary Data**

**A different view on fine-scale population structure in Western African populations**

Kridsadakorn Chaichoompu^1,2*^, Fentaw Abegaz Yazew^1^, Bruno Cavadas^3,4^, Bertram Müller-Myhsok^2^, Luísa Pereira^3,4^, and Kristel Van Steen^1,5*^

^1^ GIGA-R Medical Genomics - BIO3, University of Liege, Avenue de l'Hôpital 11, 4000 Liege, Belgium.

^2^ Max Planck Institute of Psychiatry, 80804 Munich, Germany

^3^ Instituto de Investigação e Inovação em Saúde, Universidade do Porto (i3S), Rua Alfredo Allen, 208 | 4200-135 Porto, Portugal.

^4^ Instituto de Patologia e Imunologia Molecular da Universidade do Porto (IPATIMUP), Rua Júlio Amaral de Carvalho, 45 | 4200-135 Porto, Portugal.

^5^ WELBIO (Walloon Excellence in Lifesciences and Biotechnology), Avenue Pasteur 6, 1300 Wavre, Belgium.

*corresponding authors

E-mails: kridsadakorn@biostatgen.org [KC], kristel.vansteen@uliege.ac.be [KVS]

**Supplementary Table S1** The African dataset.

| **Labels** | **Populations** | **Number of individuals** | **Sources** |
| --- | --- | --- | --- |
| ACB | African Caribbean in Barbados | 96 | 1000 Genomes |
| ASW | African ancestry in Southwest USA | 61 | 1000 Genomes |
| BGM | Gurmatche in Burkina Faso | 15 | Triska et al. 2015 |
| BGR | Gurunsi in Burkina Faso | 16 | Triska et al. 2015 |
| BM1 | Mossi I in Burkina Faso | 50 | Busby et al. 2016 |
| BM2 | Mossi II in Burkina Faso | 17 | Triska et al. 2015 |
| CBT | Bantu in Cameroon | 50 | Busby et al. 2016 |
| CSB | Semi-Bantu in Cameroon | 50 | Busby et al. 2016 |
| ESN | Esan in Nigeria | 99 | 1000 Genomes |
| GF1 | Fula I in the Gambia | 47 | Busby et al. 2016 |
| GF2 | Fula II in the Gambia | 48 | Busby et al. 2016 |
| GJL | Jola in the Gambia | 50 | Busby et al. 2016 |
| GMD | Mandinka II in the Gambia | 50 | Busby et al. 2016 |
| GMJ | Manjago in the Gambia | 47 | Busby et al. 2016 |
| GNA | Akans in Ghana | 50 | Busby et al. 2016 |
| GNK | Kasem in Ghana | 50 | Busby et al. 2016 |
| GNN | Nankam in Ghana | 50 | Busby et al. 2016 |
| GSH | Serehule in the Gambia | 47 | Busby et al. 2016 |
| GSR | Serere in the Gambia | 50 | Busby et al. 2016 |
| GWD | Gambian in Western Division – Mandinka | 113 | 1000 Genomes |
| GWL | Wollof in the Gambia | 48 | Busby et al. 2016 |
| MLB | Bambara in Mali | 50 | Busby et al. 2016 |
| MLM | Malinke in Mali | 49 | Busby et al. 2016 |
| MSL | Mende in Sierra Leone | 85 | 1000 Genomes |
| YRI | Yoruba in Ibadan, Nigeria | 108 | 1000 Genomes |

**Supplementary Table S2** Intermediate quality control results for the African dataset.

| Quality control steps | Number of Individuals | Number of SNPs |
| --- | --- | --- |
| Obtained dataset | 1,396 | 320,007 |
| Filter out non-founders | 1,396 | 320,007 |
| Filter only Chr 1-22 | 1,396 | 320,007 |
| LD pruning (r^2^<0.2) | 1,396 | 156,713 |
| HWE (0.001) | 1,396 | 155,862 |
| Call rate (95%) | 1,396 | 155,862 |
| Filter out missing SNPs (2%) | 1,396 | 155,752 |
| MAF (0.05) | 1,396 | 138,111 |

**Supplementary Table S3** Genetic distance (FST) among all ethnic groups of African dataset computed using Hudson’s method.

| ASW | 0.003 |  |  |  |  |  |  |  |  |  |  | GWD | 0.001 |  |  |  |  |  |
| --- | --- | --- | --- | --- | --- | --- | --- | --- | --- | --- | --- | --- | --- | --- | --- | --- | --- | --- |
| BGM | 0.004 | 0.010 |  |  |  |  |  |  |  |  |  | GWL | 0.000 | 0.001 |  |  |  |  |
| BGR | 0.005 | 0.011 | 0.000 |  |  |  |  |  |  |  |  | MLB | 0.003 | 0.003 | 0.003 |  |  |  |
| BM1 | 0.003 | 0.010 | 0.001 | 0.002 |  |  |  |  |  |  |  | MLM | 0.002 | 0.002 | 0.002 | 0.001 |  |  |
| BM2 | 0.005 | 0.011 | 0.001 | 0.001 | 0.001 |  |  |  |  |  |  | MSL | 0.004 | 0.004 | 0.005 | 0.002 | 0.002 |  |
| CBT | 0.005 | 0.010 | 0.005 | 0.006 | 0.005 | 0.005 |  |  |  |  |  | YRI | 0.006 | 0.006 | 0.006 | 0.003 | 0.004 | 0.004 |
| CSB | 0.004 | 0.009 | 0.004 | 0.004 | 0.003 | 0.004 | 0.001 |  |  |  |  |  | GSR | GWD | GWL | MLB | MLM | MSL |
| ESN | 0.004 | 0.010 | 0.003 | 0.004 | 0.003 | 0.004 | 0.004 | 0.002 |  |  |  |  |  |  |  |  |  |  |
| GF1 | 0.016 | 0.014 | 0.020 | 0.021 | 0.021 | 0.021 | 0.024 | 0.023 | 0.024 |  |  |  |  |  |  |  |  |  |
| GF2 | 0.004 | 0.009 | 0.003 | 0.003 | 0.002 | 0.003 | 0.005 | 0.004 | 0.005 | 0.018 |  |  |  |  |  |  |  |  |
| GJL | 0.010 | 0.015 | 0.008 | 0.009 | 0.008 | 0.008 | 0.011 | 0.010 | 0.011 | 0.022 | 0.005 |  |  |  |  |  |  |  |
| GMD | 0.006 | 0.010 | 0.005 | 0.005 | 0.004 | 0.005 | 0.008 | 0.006 | 0.007 | 0.017 | 0.001 | 0.003 |  |  |  |  |  |  |
| GMJ | 0.007 | 0.012 | 0.006 | 0.006 | 0.005 | 0.006 | 0.009 | 0.008 | 0.008 | 0.019 | 0.003 | 0.003 | 0.001 |  |  |  |  |  |
| GNA | 0.003 | 0.010 | 0.002 | 0.003 | 0.002 | 0.002 | 0.005 | 0.003 | 0.003 | 0.022 | 0.002 | 0.009 | 0.005 | 0.006 |  |  |  |  |
| GNK | 0.004 | 0.010 | 0.001 | 0.001 | 0.001 | 0.002 | 0.005 | 0.004 | 0.003 | 0.021 | 0.003 | 0.009 | 0.005 | 0.006 | 0.002 |  |  |  |
| GNN | 0.004 | 0.010 | 0.001 | 0.001 | 0.001 | 0.002 | 0.005 | 0.003 | 0.003 | 0.021 | 0.002 | 0.009 | 0.004 | 0.006 | 0.001 | 0.000 |  |  |
| GSH | 0.005 | 0.010 | 0.004 | 0.005 | 0.004 | 0.004 | 0.007 | 0.006 | 0.007 | 0.017 | 0.001 | 0.005 | 0.001 | 0.003 | 0.005 | 0.004 | 0.004 |  |
| GSR | 0.006 | 0.011 | 0.005 | 0.006 | 0.004 | 0.005 | 0.008 | 0.007 | 0.007 | 0.017 | 0.002 | 0.003 | 0.000 | 0.002 | 0.005 | 0.005 | 0.005 | 0.001 |
| GWD | 0.006 | 0.011 | 0.005 | 0.006 | 0.004 | 0.005 | 0.008 | 0.007 | 0.007 | 0.018 | 0.002 | 0.002 | 0.000 | 0.001 | 0.005 | 0.005 | 0.005 | 0.001 |
| GWL | 0.006 | 0.011 | 0.005 | 0.006 | 0.005 | 0.005 | 0.008 | 0.007 | 0.008 | 0.016 | 0.002 | 0.004 | 0.001 | 0.002 | 0.006 | 0.006 | 0.005 | 0.001 |
| MLB | 0.004 | 0.009 | 0.002 | 0.002 | 0.001 | 0.002 | 0.005 | 0.004 | 0.004 | 0.019 | 0.001 | 0.007 | 0.002 | 0.004 | 0.002 | 0.002 | 0.002 | 0.002 |
| MLM | 0.004 | 0.010 | 0.003 | 0.003 | 0.002 | 0.003 | 0.006 | 0.005 | 0.005 | 0.018 | 0.000 | 0.006 | 0.001 | 0.003 | 0.003 | 0.003 | 0.003 | 0.001 |
| MSL | 0.005 | 0.011 | 0.004 | 0.004 | 0.003 | 0.004 | 0.006 | 0.005 | 0.005 | 0.021 | 0.001 | 0.007 | 0.003 | 0.004 | 0.003 | 0.003 | 0.003 | 0.003 |
| YRI | 0.003 | 0.009 | 0.002 | 0.003 | 0.002 | 0.003 | 0.003 | 0.002 | 0.001 | 0.022 | 0.003 | 0.010 | 0.005 | 0.007 | 0.002 | 0.002 | 0.002 | 0.005 |
|  | ACB | ASW | BGM | BGR | BM1 | BM2 | CBT | CSB | ESN | GF1 | GF2 | GJL | GMD | GMJ | GNA | GNK | GNN | GSH |

**Supplementary Table S4** Clusters obtained by IPCAPS in the African dataset. Columns highlighted in grey refer to supplementary groups of individuals, which are regarded as outliers.

| **Populations** | **IPCAPS groups** | | | | | | | | | | | | | | | | | | | | | **Totals** |
| --- | --- | --- | --- | --- | --- | --- | --- | --- | --- | --- | --- | --- | --- | --- | --- | --- | --- | --- | --- | --- | --- | --- |
|  | **1** | **2** | **3** | **4** | **5** | **6** | **7** | **8** | **9** | **10** | **11** | **12** | **13** | **14** | **15** | **16** | **17** | **18** | **19** | **20** | **21** |  |
| ACB | 0 | 0 | 0 | 0 | 0 | 0 | 10 | 0 | 0 | 39 | 41 | 4 | 0 | 0 | 0 | 0 | 0 | 0 | 0 | 0 | 2 | 96 |
| ASW | 0 | 0 | 0 | 0 | 0 | 0 | 1 | 0 | 0 | 4 | 24 | 19 | 0 | 2 | 4 | 0 | 2 | 2 | 0 | 3 | 0 | 61 |
| BGM | 0 | 0 | 0 | 0 | 0 | 15 | 0 | 0 | 0 | 0 | 0 | 0 | 0 | 0 | 0 | 0 | 0 | 0 | 0 | 0 | 0 | 15 |
| BGR | 0 | 0 | 0 | 0 | 0 | 16 | 0 | 0 | 0 | 0 | 0 | 0 | 0 | 0 | 0 | 0 | 0 | 0 | 0 | 0 | 0 | 16 |
| BM1 | 0 | 0 | 0 | 0 | 0 | 50 | 0 | 0 | 0 | 0 | 0 | 0 | 0 | 0 | 0 | 0 | 0 | 0 | 0 | 0 | 0 | 50 |
| BM2 | 0 | 0 | 0 | 0 | 0 | 17 | 0 | 0 | 0 | 0 | 0 | 0 | 0 | 0 | 0 | 0 | 0 | 0 | 0 | 0 | 0 | 17 |
| CBT | 0 | 0 | 0 | 0 | 0 | 0 | 0 | 48 | 0 | 0 | 0 | 0 | 0 | 0 | 0 | 0 | 0 | 0 | 2 | 0 | 0 | 50 |
| CSB | 0 | 0 | 0 | 0 | 0 | 0 | 1 | 0 | 49 | 0 | 0 | 0 | 0 | 0 | 0 | 0 | 0 | 0 | 0 | 0 | 0 | 50 |
| ESN | 0 | 0 | 0 | 0 | 0 | 0 | 94 | 0 | 0 | 0 | 0 | 0 | 2 | 0 | 0 | 3 | 0 | 0 | 0 | 0 | 0 | 99 |
| GF1 | 47 | 0 | 0 | 0 | 0 | 0 | 0 | 0 | 0 | 0 | 0 | 0 | 0 | 0 | 0 | 0 | 0 | 0 | 0 | 0 | 0 | 47 |
| GF2 | 0 | 3 | 43 | 2 | 0 | 0 | 0 | 0 | 0 | 0 | 0 | 0 | 0 | 0 | 0 | 0 | 0 | 0 | 0 | 0 | 0 | 48 |
| GJL | 0 | 0 | 0 | 0 | 50 | 0 | 0 | 0 | 0 | 0 | 0 | 0 | 0 | 0 | 0 | 0 | 0 | 0 | 0 | 0 | 0 | 50 |
| GMD | 0 | 0 | 0 | 50 | 0 | 0 | 0 | 0 | 0 | 0 | 0 | 0 | 0 | 0 | 0 | 0 | 0 | 0 | 0 | 0 | 0 | 50 |
| GMJ | 0 | 0 | 2 | 42 | 1 | 0 | 0 | 0 | 0 | 0 | 0 | 2 | 0 | 0 | 0 | 0 | 0 | 0 | 0 | 0 | 0 | 47 |
| GNA | 0 | 0 | 0 | 0 | 0 | 50 | 0 | 0 | 0 | 0 | 0 | 0 | 0 | 0 | 0 | 0 | 0 | 0 | 0 | 0 | 0 | 50 |
| GNK | 0 | 0 | 0 | 0 | 0 | 50 | 0 | 0 | 0 | 0 | 0 | 0 | 0 | 0 | 0 | 0 | 0 | 0 | 0 | 0 | 0 | 50 |
| GNN | 0 | 0 | 0 | 0 | 0 | 50 | 0 | 0 | 0 | 0 | 0 | 0 | 0 | 0 | 0 | 0 | 0 | 0 | 0 | 0 | 0 | 50 |
| GSH | 0 | 0 | 7 | 40 | 0 | 0 | 0 | 0 | 0 | 0 | 0 | 0 | 0 | 0 | 0 | 0 | 0 | 0 | 0 | 0 | 0 | 47 |
| GSR | 0 | 0 | 3 | 47 | 0 | 0 | 0 | 0 | 0 | 0 | 0 | 0 | 0 | 0 | 0 | 0 | 0 | 0 | 0 | 0 | 0 | 50 |
| GWD | 0 | 0 | 3 | 108 | 1 | 0 | 0 | 0 | 0 | 0 | 1 | 0 | 0 | 0 | 0 | 0 | 0 | 0 | 0 | 0 | 0 | 113 |
| GWL | 0 | 0 | 1 | 47 | 0 | 0 | 0 | 0 | 0 | 0 | 0 | 0 | 0 | 0 | 0 | 0 | 0 | 0 | 0 | 0 | 0 | 48 |
| MLB | 0 | 0 | 50 | 0 | 0 | 0 | 0 | 0 | 0 | 0 | 0 | 0 | 0 | 0 | 0 | 0 | 0 | 0 | 0 | 0 | 0 | 50 |
| MLM | 0 | 0 | 45 | 3 | 0 | 0 | 0 | 0 | 0 | 1 | 0 | 0 | 0 | 0 | 0 | 0 | 0 | 0 | 0 | 0 | 0 | 49 |
| MSL | 0 | 84 | 1 | 0 | 0 | 0 | 0 | 0 | 0 | 0 | 0 | 0 | 0 | 0 | 0 | 0 | 0 | 0 | 0 | 0 | 0 | 85 |
| YRI | 0 | 0 | 0 | 0 | 0 | 1 | 107 | 0 | 0 | 0 | 0 | 0 | 0 | 0 | 0 | 0 | 0 | 0 | 0 | 0 | 0 | 108 |
| **Totals** | 47 | 87 | 155 | 339 | 52 | 249 | 213 | 48 | 49 | 44 | 66 | 25 | 2 | 2 | 4 | 3 | 2 | 2 | 2 | 3 | 2 | 1396 |
